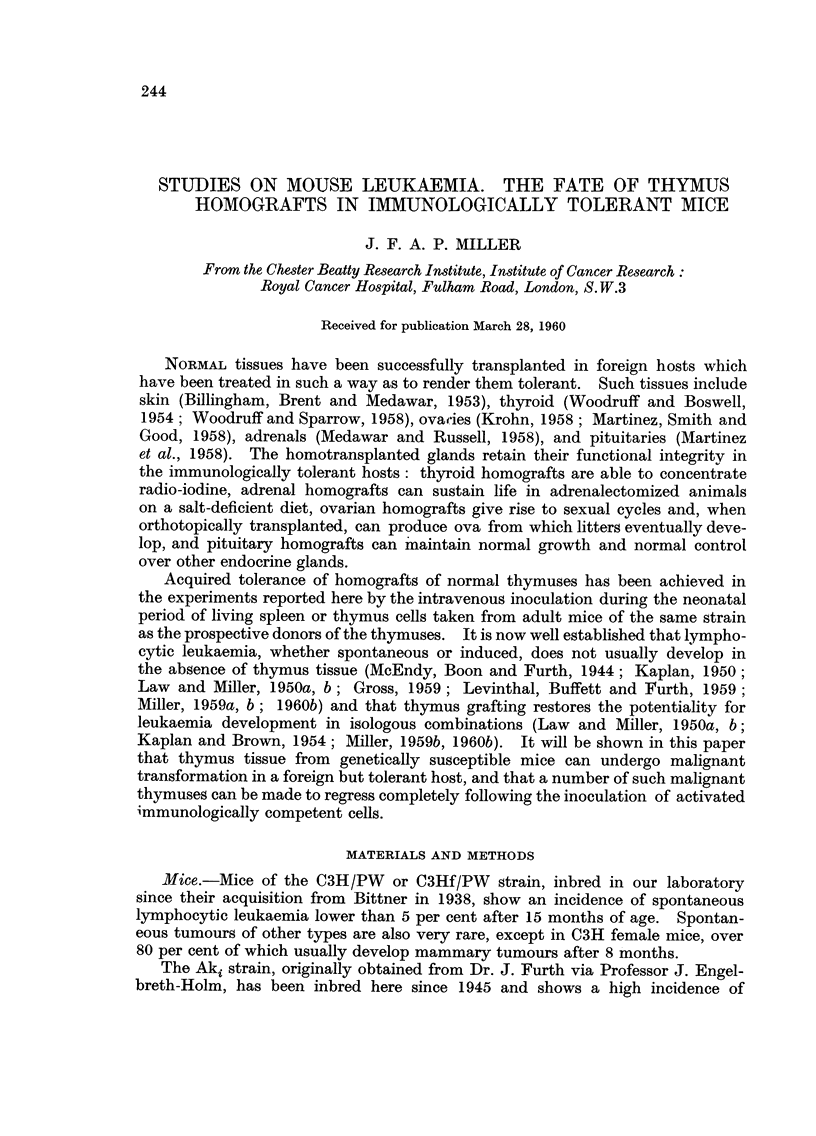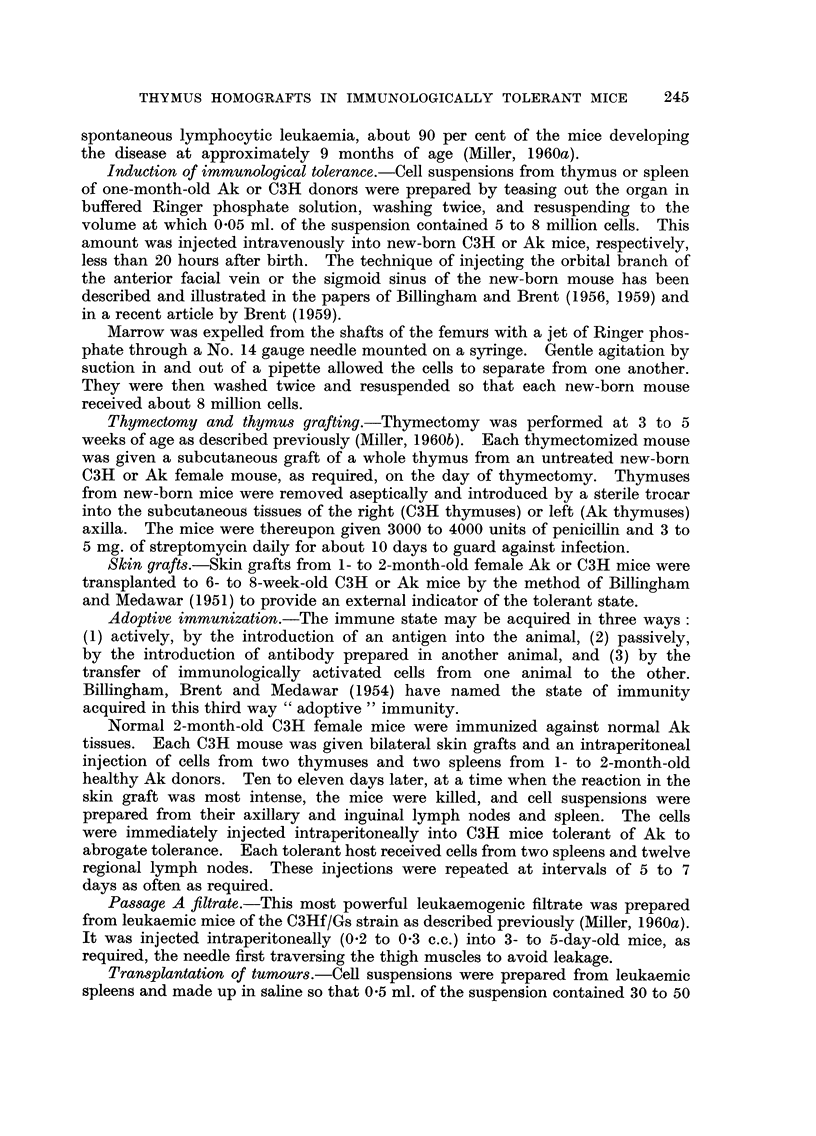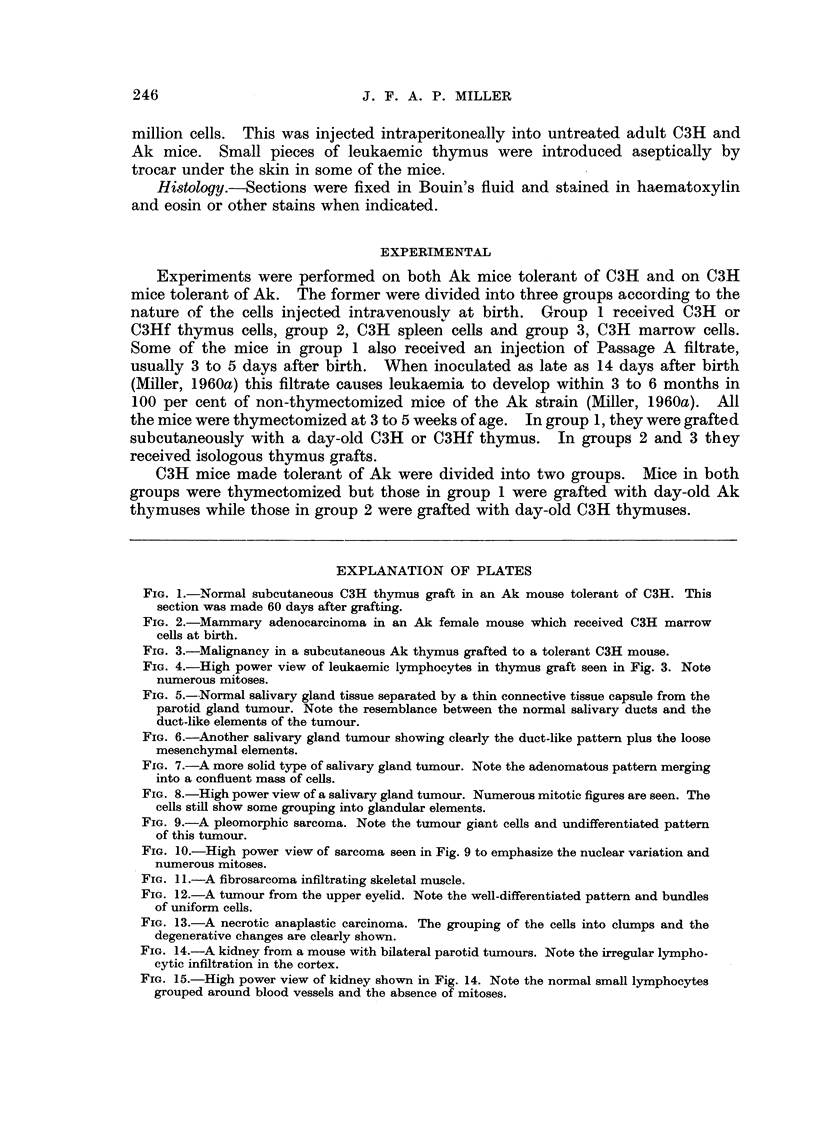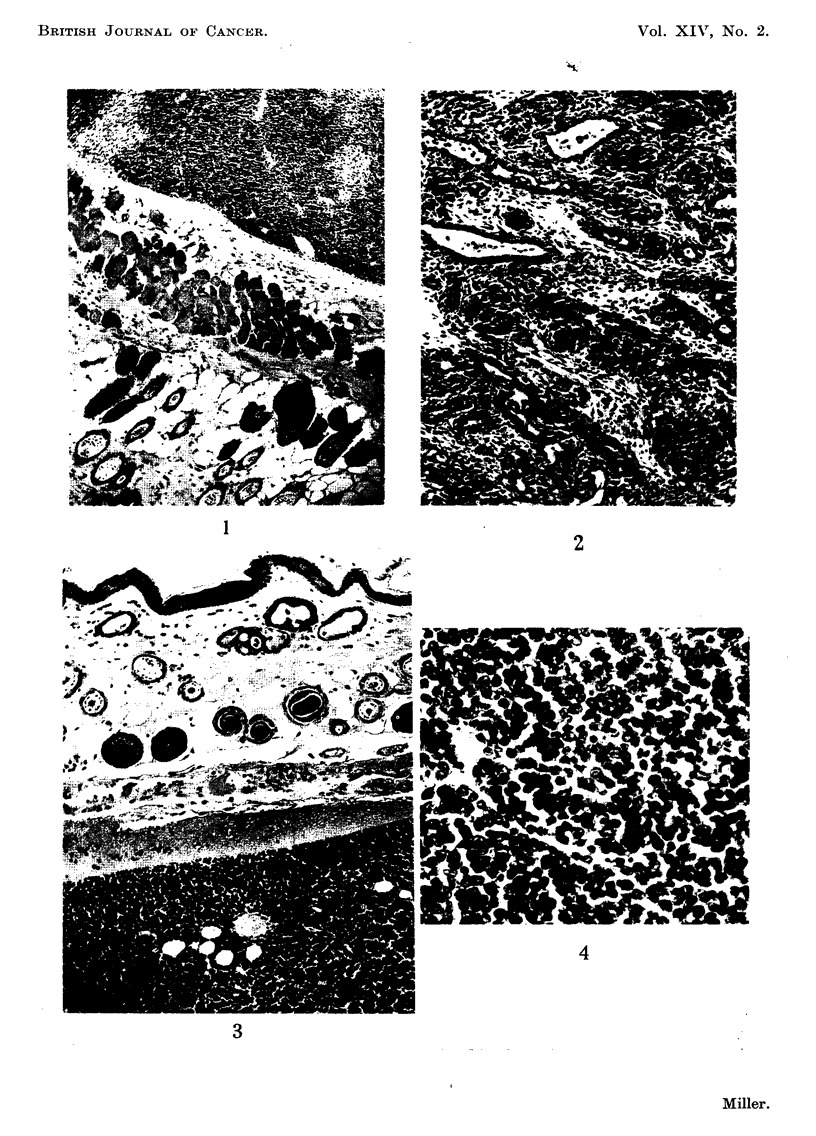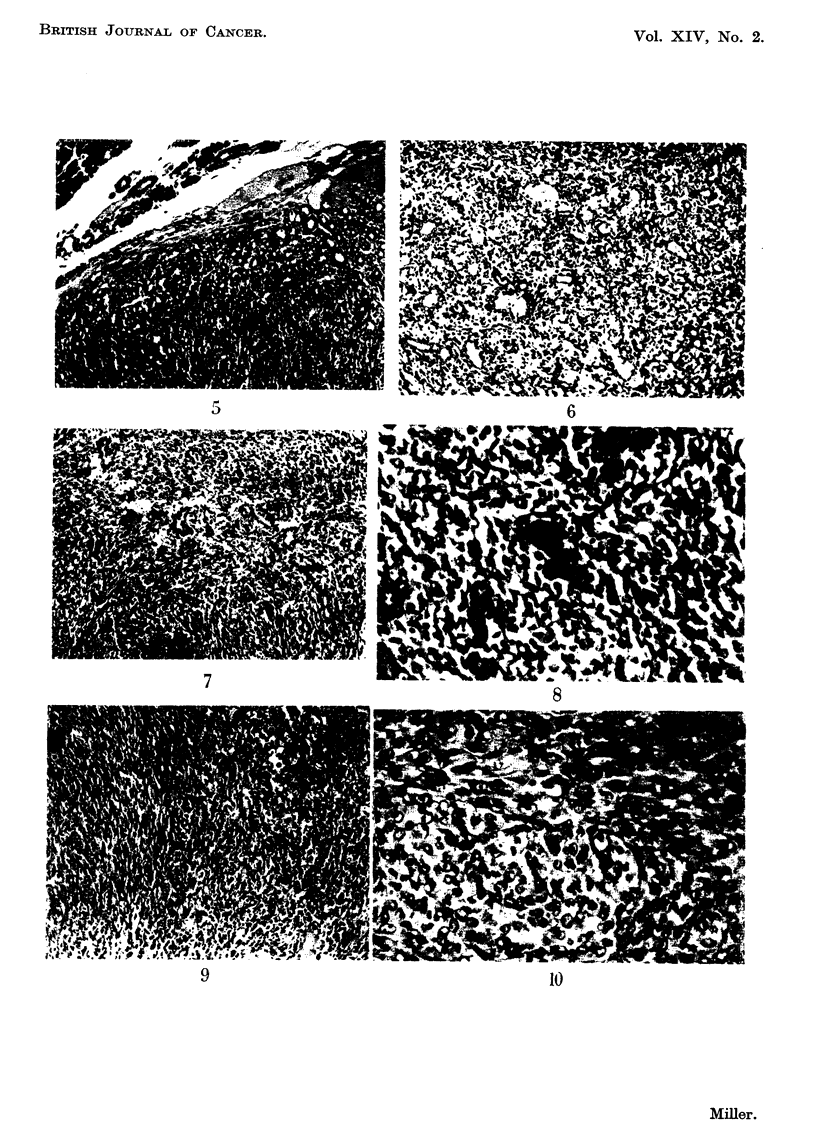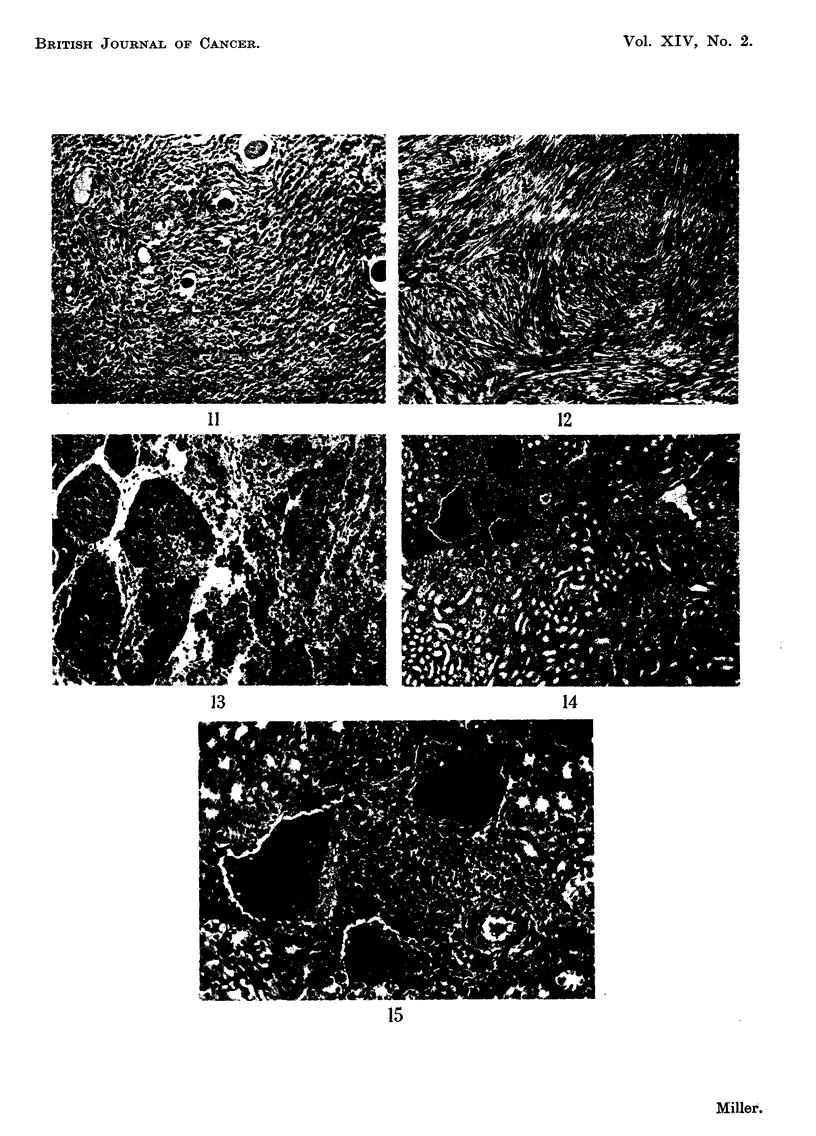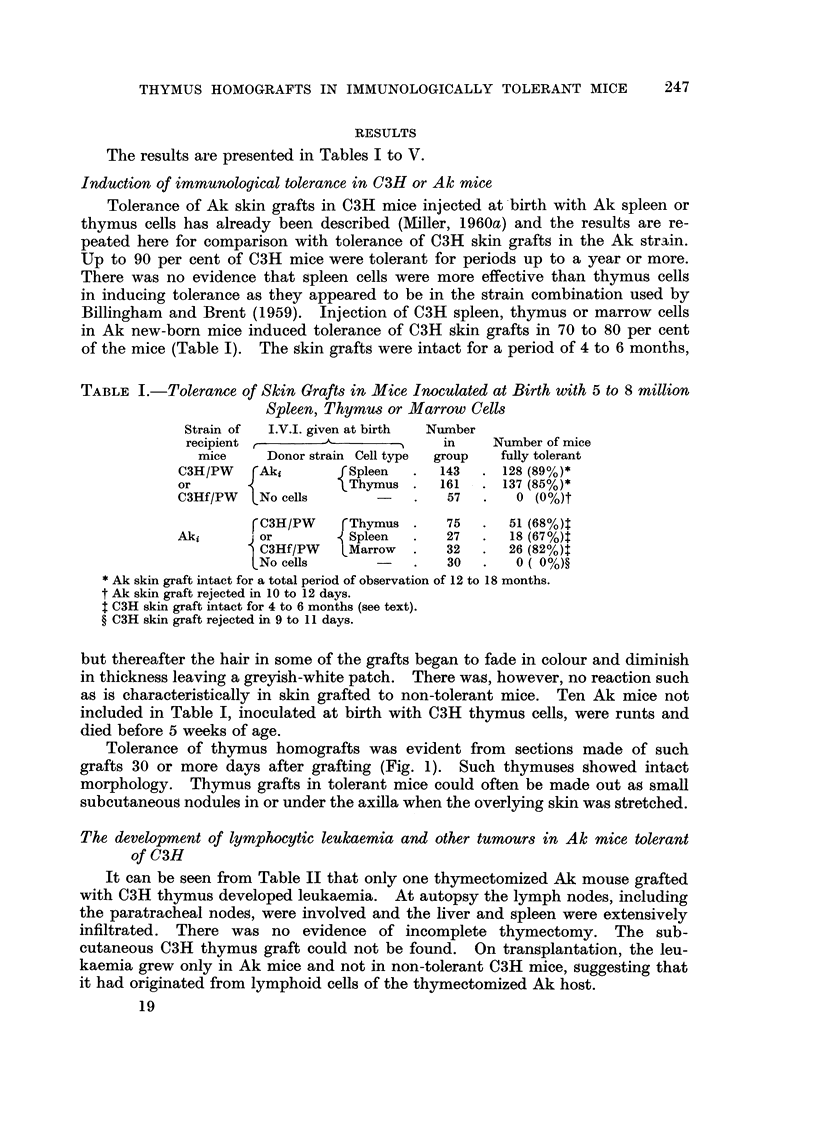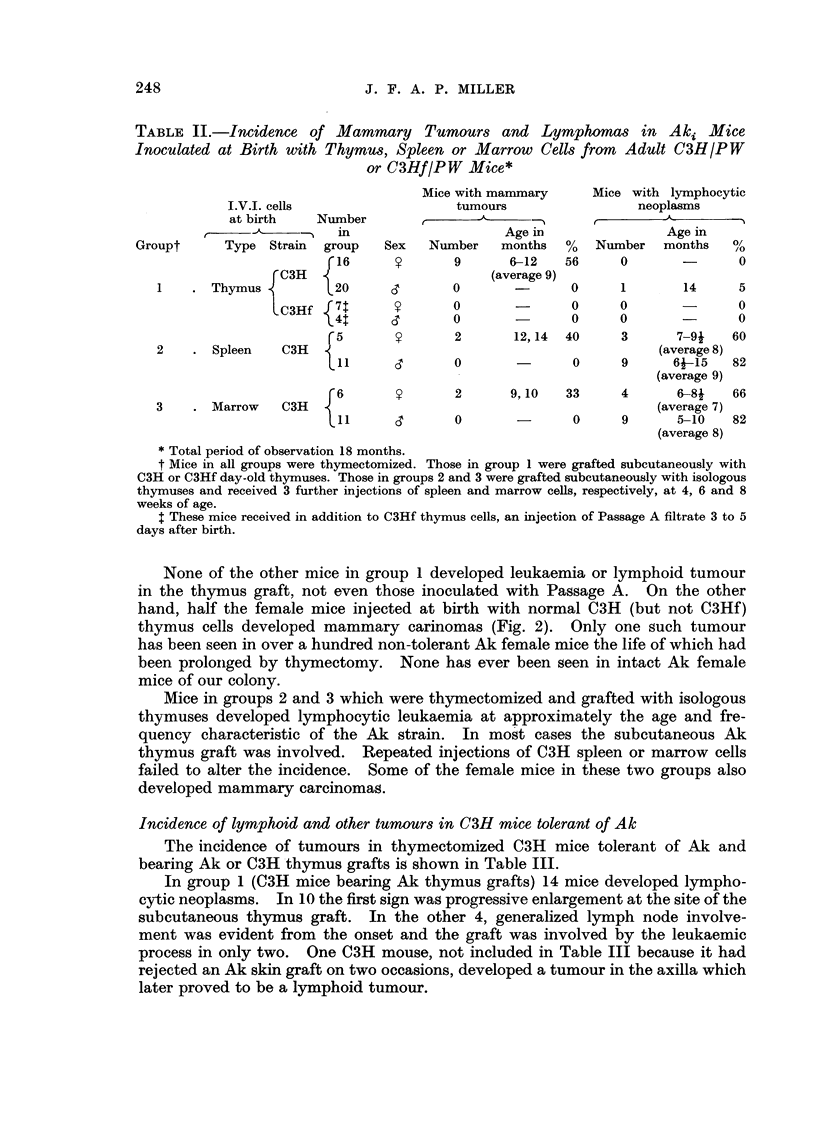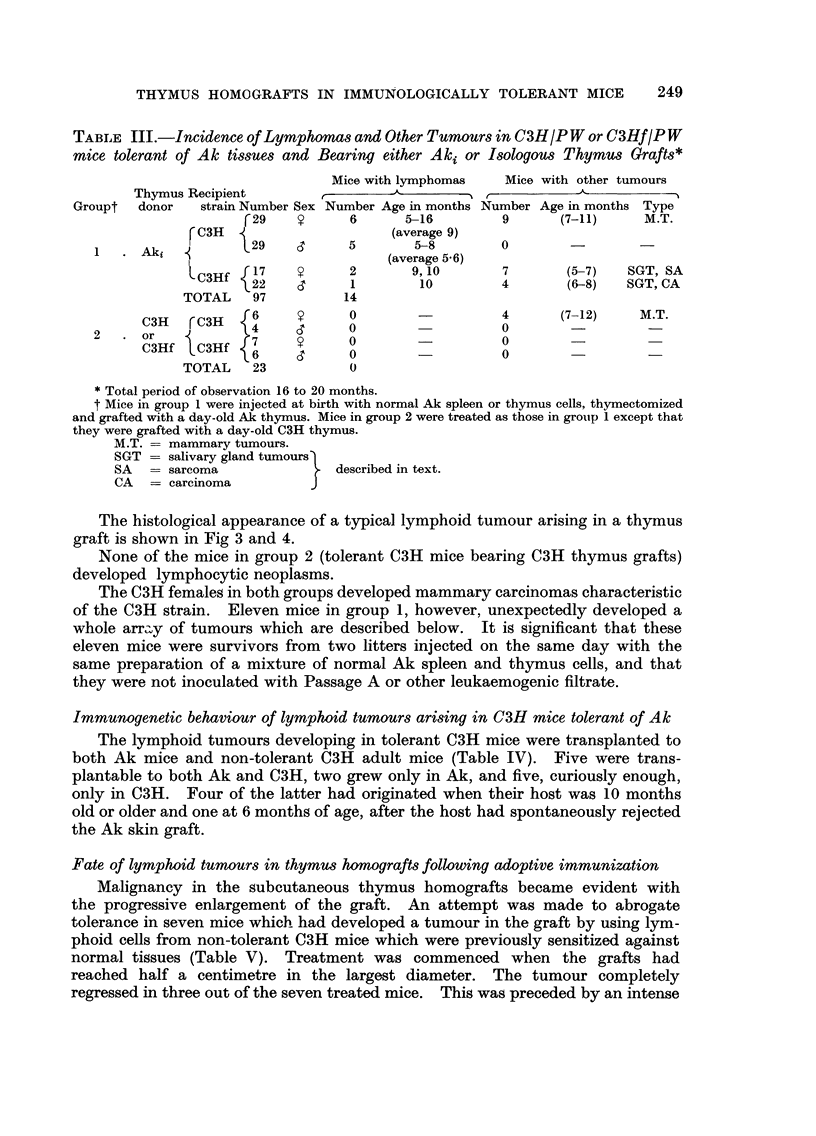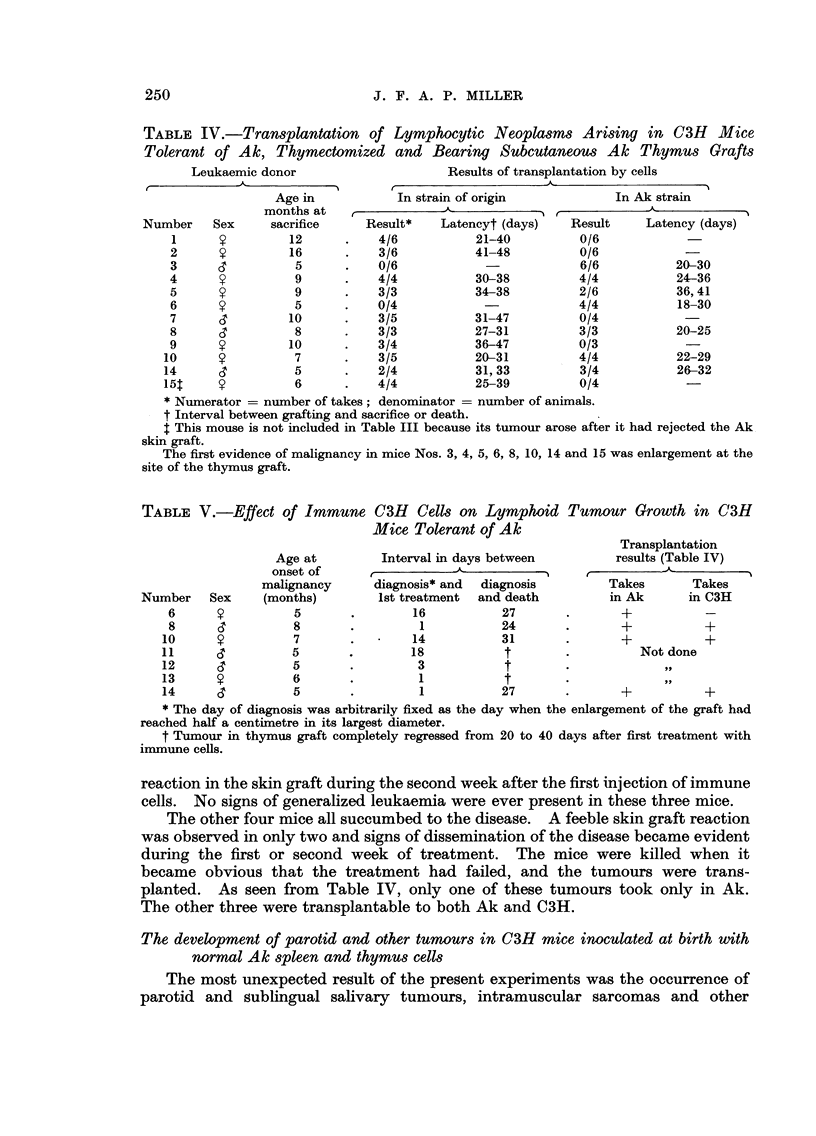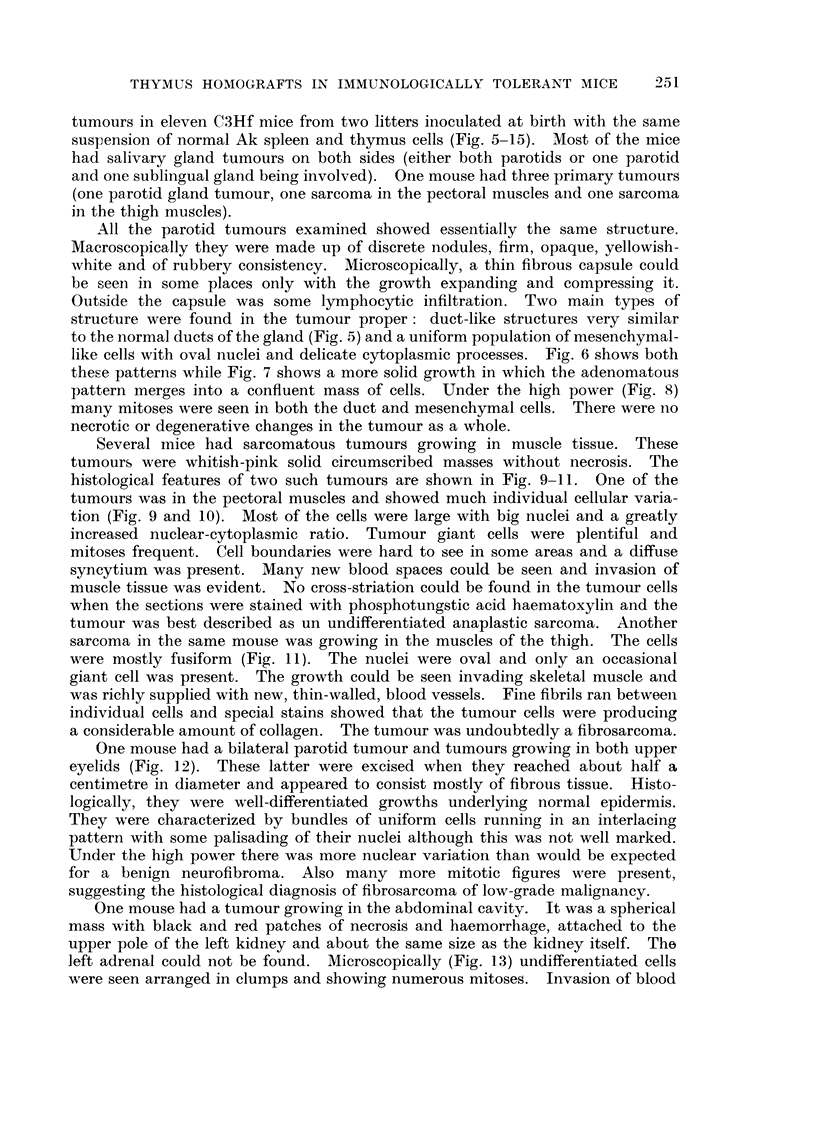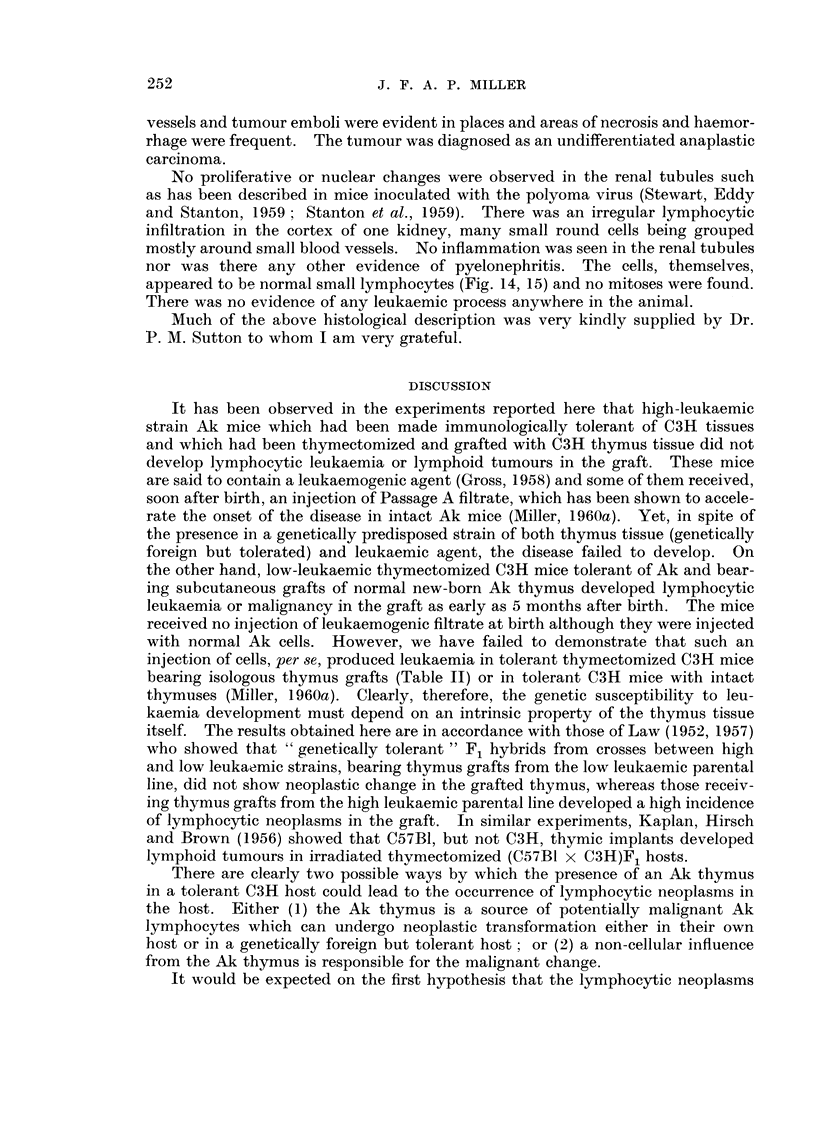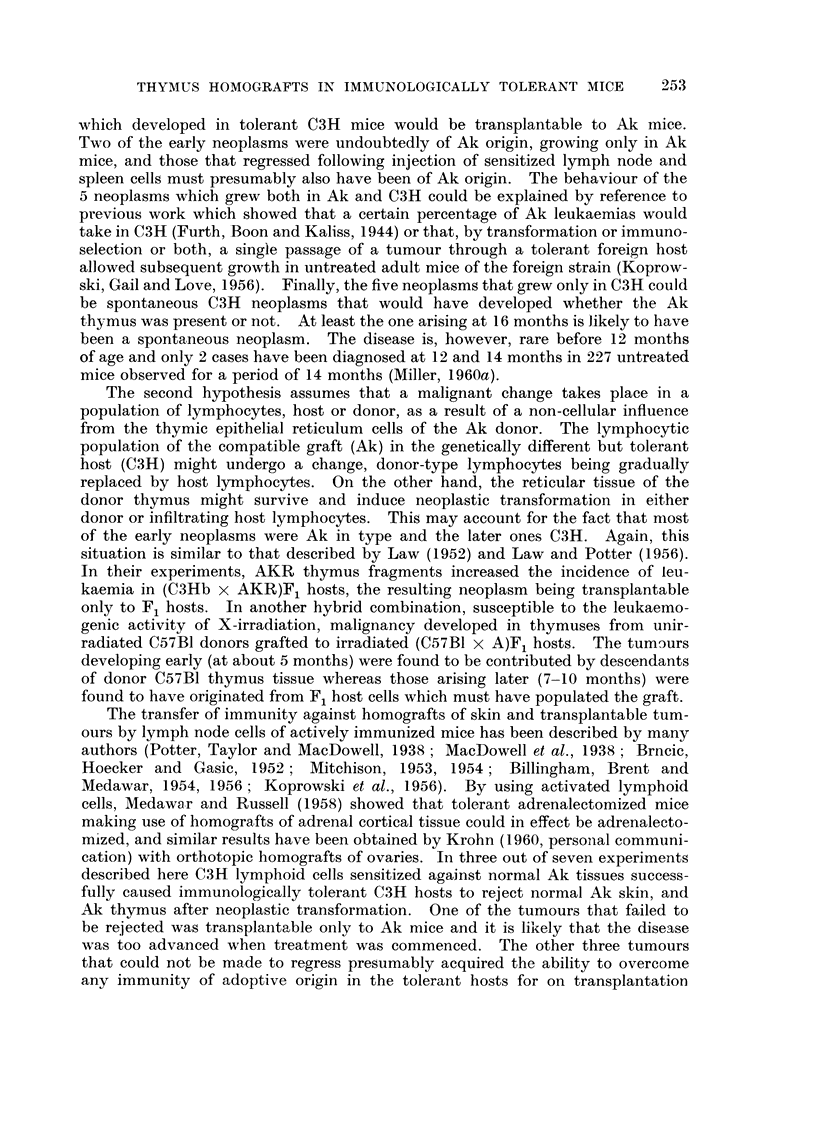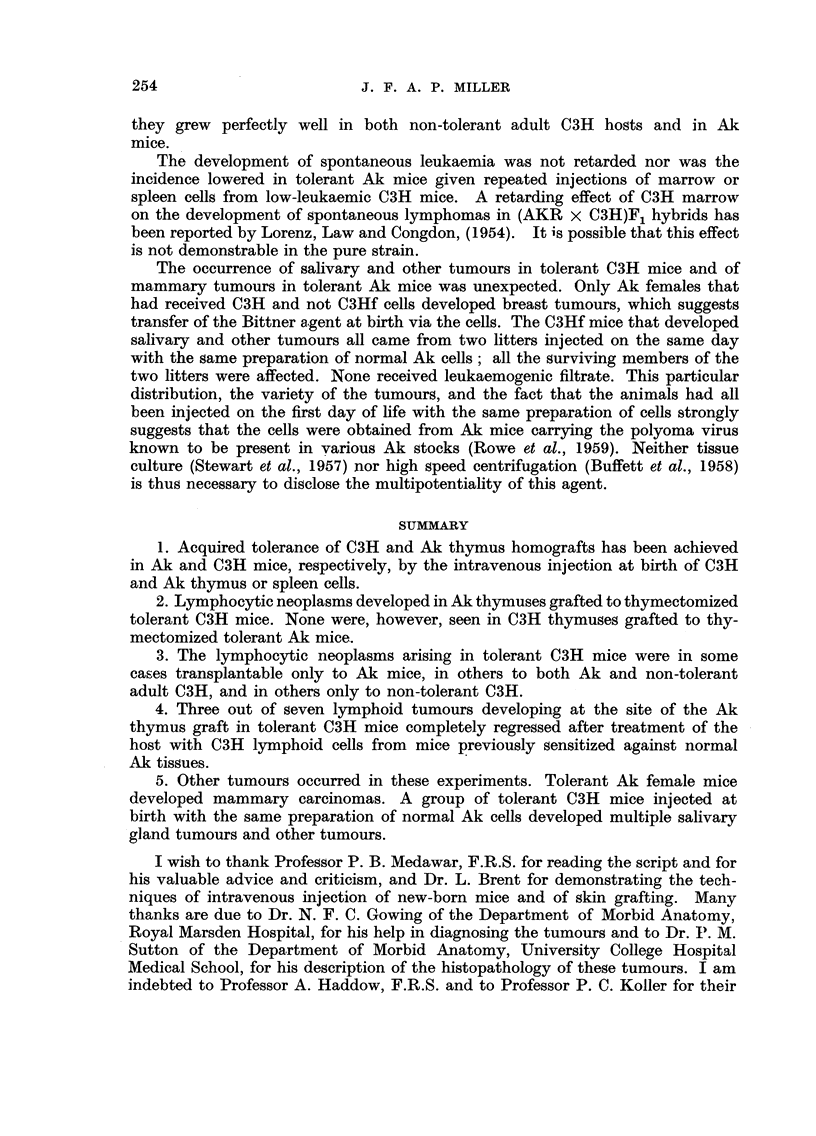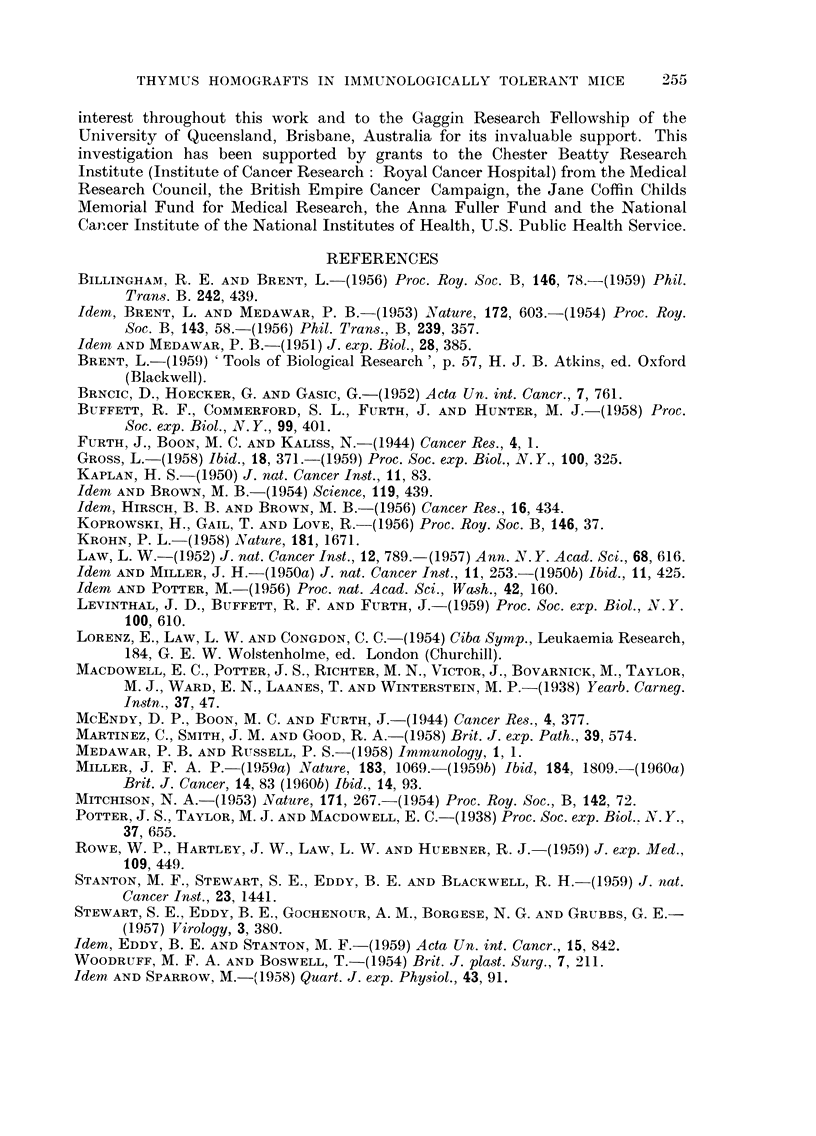# Studies on Mouse Leukaemia. The Fate of Thymus Homografts in Immunologically Tolerant Mice

**DOI:** 10.1038/bjc.1960.29

**Published:** 1960-06

**Authors:** J. F. A. P. Miller

## Abstract

**Images:**


### STUDIES ON MOUSE LEUKAEMIA. THE FATE OF THYMUS HOMOGRAFTS IN IMMUNOLOGICALLY TOLERANT MICE

J. F. A. P. MILLER

*From the Chester Beatty Research Institute, Institute of Cancer Research : Royal Cancer Hospital, Fulham Road, London, S.W.3*

Received for publication March 28, 1960

NORMAL tissues have been successfully transplanted in foreign hosts which have been treated in such a way as to render them tolerant. Such tissues include skin (Billingham, Brent and Medawar, 1953), thyroid (Woodruff and Boswell, 1954 ; Woodruff and Sparrow, 1958), ovaries (Krohn, 1958 ; Martinez, Smith and Good, 1958), adrenals (Medawar and Russell, 1958), and pituitaries (Martinez *et al.*, 1958). The homotransplanted glands retain their functional integrity in the immunologically tolerant hosts : thyroid homografts are able to concentrate radio-iodine, adrenal homografts can sustain life in adrenalectomized animals on a salt-deficient diet, ovarian homografts give rise to sexual cycles and, when orthotopically transplanted, can produce ova from which litters eventually develop, and pituitary homografts can maintain normal growth and normal control over other endocrine glands.

Acquired tolerance of homografts of normal thymuses has been achieved in the experiments reported here by the intravenous inoculation during the neonatal period of living spleen or thymus cells taken from adult mice of the same strain as the prospective donors of the thymuses. It is now well established that lymphocytic leukaemia, whether spontaneous or induced, does not usually develop in the absence of thymus tissue (McEndy, Boon and Furth, 1944 ; Kaplan, 1950 ; Law and Miller, 1950*a*, *b* ; Gross, 1959 ; Levinthal, Buffett and Furth, 1959 ; Miller, 1959*a*, *b* ; 1960*b*) and that thymus grafting restores the potentiality for leukaemia development in isologous combinations (Law and Miller, 1950*a*, *b* ; Kaplan and Brown, 1954 ; Miller, 1959*b*, 1960*b*). It will be shown in this paper that thymus tissue from genetically susceptible mice can undergo malignant transformation in a foreign but tolerant host, and that a number of such malignant thymuses can be made to regress completely following the inoculation of activated immunologically competent cells.

### MATERIALS AND METHODS

*Mice.*—Mice of the C3H/PW or C3Hf/PW strain, inbred in our laboratory since their acquisition from Bittner in 1938, show an incidence of spontaneous lymphocytic leukaemia lower than 5 per cent after 15 months of age. Spontaneous tumours of other types are also very rare, except in C3H female mice, over 80 per cent of which usually develop mammary tumours after 8 months.

The $Ak_i$ strain, originally obtained from Dr. J. Furth via Professor J. Engelbreth-Holm, has been inbred here since 1945 and shows a high incidence of spontaneous lymphocytic leukaemia, about 90 per cent of the mice developing the disease at approximately 9 months of age (Miller, 1960*a*).

*Induction of immunological tolerance.*—Cell suspensions from thymus or spleen of one-month-old Ak or C3H donors were prepared by teasing out the organ in buffered Ringer phosphate solution, washing twice, and resuspending to the volume at which 0·05 ml. of the suspension contained 5 to 8 million cells. This amount was injected intravenously into new-born C3H or Ak mice, respectively, less than 20 hours after birth. The technique of injecting the orbital branch of the anterior facial vein or the sigmoid sinus of the new-born mouse has been described and illustrated in the papers of Billingham and Brent (1956, 1959) and in a recent article by Brent (1959).

Marrow was expelled from the shafts of the femurs with a jet of Ringer phosphate through a No. 14 gauge needle mounted on a syringe. Gentle agitation by suction in and out of a pipette allowed the cells to separate from one another. They were then washed twice and resuspended so that each new-born mouse received about 8 million cells.

*Thymectomy and thymus grafting.*—Thymectomy was performed at 3 to 5 weeks of age as described previously (Miller, 1960*b*). Each thymectomized mouse was given a subcutaneous graft of a whole thymus from an untreated new-born C3H or Ak female mouse, as required, on the day of thymectomy. Thymuses from new-born mice were removed aseptically and introduced by a sterile trocar into the subcutaneous tissues of the right (C3H thymuses) or left (Ak thymuses) axilla. The mice were thereupon given 3000 to 4000 units of penicillin and 3 to 5 mg. of streptomycin daily for about 10 days to guard against infection.

*Skin grafts.*—Skin grafts from 1- to 2-month-old female Ak or C3H mice were transplanted to 6- to 8-week-old C3H or Ak mice by the method of Billingham and Medawar (1951) to provide an external indicator of the tolerant state.

*Adoptive immunization.*—The immune state may be acquired in three ways : (1) actively, by the introduction of an antigen into the animal, (2) passively, by the introduction of antibody prepared in another animal, and (3) by the transfer of immunologically activated cells from one animal to the other. Billingham, Brent and Medawar (1954) have named the state of immunity acquired in this third way " adoptive " immunity.

Normal 2-month-old C3H female mice were immunized against normal Ak tissues. Each C3H mouse was given bilateral skin grafts and an intraperitoneal injection of cells from two thymuses and two spleens from 1- to 2-month-old healthy Ak donors. Ten to eleven days later, at a time when the reaction in the skin graft was most intense, the mice were killed, and cell suspensions were prepared from their axillary and inguinal lymph nodes and spleen. The cells were immediately injected intraperitoneally into C3H mice tolerant of Ak to abrogate tolerance. Each tolerant host received cells from two spleens and twelve regional lymph nodes. These injections were repeated at intervals of 5 to 7 days as often as required.

*Passage A filtrate.*—This most powerful leukaemogenic filtrate was prepared from leukaemic mice of the C3Hf/Gs strain as described previously (Miller, 1960*a*). It was injected intraperitoneally (0·2 to 0·3 c.c.) into 3- to 5-day-old mice, as required, the needle first traversing the thigh muscles to avoid leakage.

*Transplantation of tumours.*—Cell suspensions were prepared from leukaemic spleens and made up in saline so that 0·5 ml. of the suspension contained 30 to 50

million cells. This was injected intraperitoneally into untreated adult C3H and Ak mice. Small pieces of leukaemic thymus were introduced aseptically by trocar under the skin in some of the mice.

*Histology.*—Sections were fixed in Bouin's fluid and stained in haematoxylin and eosin or other stains when indicated.

### EXPERIMENTAL

Experiments were performed on both Ak mice tolerant of C3H and on C3H mice tolerant of Ak. The former were divided into three groups according to the nature of the cells injected intravenously at birth. Group 1 received C3H or C3Hf thymus cells, group 2, C3H spleen cells and group 3, C3H marrow cells. Some of the mice in group 1 also received an injection of Passage A filtrate, usually 3 to 5 days after birth. When inoculated as late as 14 days after birth (Miller, 1960*a*) this filtrate causes leukaemia to develop within 3 to 6 months in 100 per cent of non-thymectomized mice of the Ak strain (Miller, 1960*a*). All the mice were thymectomized at 3 to 5 weeks of age. In group 1, they were grafted subcutaneously with a day-old C3H or C3Hf thymus. In groups 2 and 3 they received isologous thymus grafts.

C3H mice made tolerant of Ak were divided into two groups. Mice in both groups were thymectomized but those in group 1 were grafted with day-old Ak thymuses while those in group 2 were grafted with day-old C3H thymuses.

---

### EXPLANATION OF PLATES

FIG. 1.—Normal subcutaneous C3H thymus graft in an Ak mouse tolerant of C3H. This section was made 60 days after grafting.

FIG. 2.—Mammary adenocarcinoma in an Ak female mouse which received C3H marrow cells at birth.

FIG. 3.—Malignancy in a subcutaneous Ak thymus grafted to a tolerant C3H mouse.

FIG. 4.—High power view of leukaemic lymphocytes in thymus graft seen in Fig. 3. Note numerous mitoses.

FIG. 5.—Normal salivary gland tissue separated by a thin connective tissue capsule from the parotid gland tumour. Note the resemblance between the normal salivary ducts and the duct-like elements of the tumour.

FIG. 6.—Another salivary gland tumour showing clearly the duct-like pattern plus the loose mesenchymal elements.

FIG. 7.—A more solid type of salivary gland tumour. Note the adenomatous pattern merging into a confluent mass of cells.

FIG. 8.—High power view of a salivary gland tumour. Numerous mitotic figures are seen. The cells still show some grouping into glandular elements.

FIG. 9.—A pleomorphic sarcoma. Note the tumour giant cells and undifferentiated pattern of this tumour.

FIG. 10.—High power view of sarcoma seen in Fig. 9 to emphasize the nuclear variation and numerous mitoses.

FIG. 11.—A fibrosarcoma infiltrating skeletal muscle.

FIG. 12.—A tumour from the upper eyelid. Note the well-differentiated pattern and bundles of uniform cells.

FIG. 13.—A necrotic anaplastic carcinoma. The grouping of the cells into clumps and the degenerative changes are clearly shown.

FIG. 14.—A kidney from a mouse with bilateral parotid tumours. Note the irregular lymphocytic infiltration in the cortex.

FIG. 15.—High power view of kidney shown in Fig. 14. Note the normal small lymphocytes grouped around blood vessels and the absence of mitoses.

1

2

3

4

5

6

7

8

9

10

Miller.

11

12

13

14

15

### RESULTS

The results are presented in Tables I to V.

*Induction of immunological tolerance in C3H or Ak mice*

Tolerance of Ak skin grafts in C3H mice injected at birth with Ak spleen or thymus cells has already been described (Miller, 1960*a*) and the results are repeated here for comparison with tolerance of C3H skin grafts in the Ak strain. Up to 90 per cent of C3H mice were tolerant for periods up to a year or more. There was no evidence that spleen cells were more effective than thymus cells in inducing tolerance as they appeared to be in the strain combination used by Billingham and Brent (1959). Injection of C3H spleen, thymus or marrow cells in Ak new-born mice induced tolerance of C3H skin grafts in 70 to 80 per cent of the mice (Table I). The skin grafts were intact for a period of 4 to 6 months,

TABLE I.—*Tolerance of Skin Grafts in Mice Inoculated at Birth with* 5 *to* 8 *million Spleen, Thymus or Marrow Cells*

| Strain of recipient mice | I.V.I. given at birth: Donor strain | I.V.I. given at birth: Cell type | Number in group | Number of mice fully tolerant |
|---|---|---|---|---|
| C3H/PW or C3Hf/PW | $Ak_i$ | Spleen | 143 | 128 (89%)* |
| | $Ak_i$ | Thymus | 161 | 137 (85%)* |
| | No cells | — | 57 | 0 (0%)† |
| $Ak_i$ | C3H/PW or C3Hf/PW | Thymus | 75 | 51 (68%)‡ |
| | | Spleen | 27 | 18 (67%)‡ |
| | | Marrow | 32 | 26 (82%)‡ |
| | No cells | — | 30 | 0 ( 0%)§ |

* Ak skin graft intact for a total period of observation of 12 to 18 months.
† Ak skin graft rejected in 10 to 12 days.
‡ C3H skin graft intact for 4 to 6 months (see text).
§ C3H skin graft rejected in 9 to 11 days.

but thereafter the hair in some of the grafts began to fade in colour and diminish in thickness leaving a greyish-white patch. There was, however, no reaction such as is characteristically in skin grafted to non-tolerant mice. Ten Ak mice not included in Table I, inoculated at birth with C3H thymus cells, were runts and died before 5 weeks of age.

Tolerance of thymus homografts was evident from sections made of such grafts 30 or more days after grafting (Fig. 1). Such thymuses showed intact morphology. Thymus grafts in tolerant mice could often be made out as small subcutaneous nodules in or under the axilla when the overlying skin was stretched.

*The development of lymphocytic leukaemia and other tumours in Ak mice tolerant of C3H*

It can be seen from Table II that only one thymectomized Ak mouse grafted with C3H thymus developed leukaemia. At autopsy the lymph nodes, including the paratracheal nodes, were involved and the liver and spleen were extensively infiltrated. There was no evidence of incomplete thymectomy. The subcutaneous C3H thymus graft could not be found. On transplantation, the leukaemia grew only in Ak mice and not in non-tolerant C3H mice, suggesting that it had originated from lymphoid cells of the thymectomized Ak host.

TABLE II.—*Incidence of Mammary Tumours and Lymphomas in $Ak_i$ Mice Inoculated at Birth with Thymus, Spleen or Marrow Cells from Adult C3H/PW or C3Hf/PW Mice**

| Group† | I.V.I. cells at birth: Type | Strain | Number in group | Sex | Mice with mammary tumours: Number | Age in months | % | Mice with lymphocytic neoplasms: Number | Age in months | % |
|---|---|---|---|---|---|---|---|---|---|---|
| 1 . | Thymus | C3H | 16 | ♀ | 9 | 6–12 (average 9) | 56 | 0 | — | 0 |
| | | | 20 | ♂ | 0 | — | 0 | 1 | 14 | 5 |
| | | C3Hf | 7‡ | ♀ | 0 | — | 0 | 0 | — | 0 |
| | | | 4‡ | ♂ | 0 | — | 0 | 0 | — | 0 |
| 2 . | Spleen | C3H | 5 | ♀ | 2 | 12, 14 | 40 | 3 | 7–9½ (average 8) | 60 |
| | | | 11 | ♂ | 0 | — | 0 | 9 | 6½–15 (average 9) | 82 |
| 3 . | Marrow | C3H | 6 | ♀ | 2 | 9, 10 | 33 | 4 | 6–8½ (average 7) | 66 |
| | | | 11 | ♂ | 0 | — | 0 | 9 | 5–10 (average 8) | 82 |

* Total period of observation 18 months.

† Mice in all groups were thymectomized. Those in group 1 were grafted subcutaneously with C3H or C3Hf day-old thymuses. Those in groups 2 and 3 were grafted subcutaneously with isologous thymuses and received 3 further injections of spleen and marrow cells, respectively, at 4, 6 and 8 weeks of age.

‡ These mice received in addition to C3Hf thymus cells, an injection of Passage A filtrate 3 to 5 days after birth.

None of the other mice in group 1 developed leukaemia or lymphoid tumour in the thymus graft, not even those inoculated with Passage A. On the other hand, half the female mice injected at birth with normal C3H (but not C3Hf) thymus cells developed mammary carinomas (Fig. 2). Only one such tumour has been seen in over a hundred non-tolerant Ak female mice the life of which had been prolonged by thymectomy. None has ever been seen in intact Ak female mice of our colony.

Mice in groups 2 and 3 which were thymectomized and grafted with isologous thymuses developed lymphocytic leukaemia at approximately the age and frequency characteristic of the Ak strain. In most cases the subcutaneous Ak thymus graft was involved. Repeated injections of C3H spleen or marrow cells failed to alter the incidence. Some of the female mice in these two groups also developed mammary carcinomas.

*Incidence of lymphoid and other tumours in C3H mice tolerant of Ak*

The incidence of tumours in thymectomized C3H mice tolerant of Ak and bearing Ak or C3H thymus grafts is shown in Table III.

In group 1 (C3H mice bearing Ak thymus grafts) 14 mice developed lymphocytic neoplasms. In 10 the first sign was progressive enlargement at the site of the subcutaneous thymus graft. In the other 4, generalized lymph node involvement was evident from the onset and the graft was involved by the leukaemic process in only two. One C3H mouse, not included in Table III because it had rejected an Ak skin graft on two occasions, developed a tumour in the axilla which later proved to be a lymphoid tumour.

TABLE III.—*Incidence of Lymphomas and Other Tumours in C3H/PW or C3Hf/PW mice tolerant of Ak tissues and Bearing either $Ak_i$ or Isologous Thymus Grafts**

| Group† | Thymus donor | Recipient strain | Number | Sex | Mice with lymphomas: Number | Age in months | Mice with other tumours: Number | Age in months | Type |
|---|---|---|---|---|---|---|---|---|---|
| 1 | $Ak_i$ | C3H | 29 | ♀ | 6 | 5–16 (average 9) | 9 | (7–11) | M.T. |
| | | | 29 | ♂ | 5 | 5–8 (average 5·6) | 0 | — | — |
| | | C3Hf | 17 | ♀ | 2 | 9, 10 | 7 | (5–7) | SGT, SA |
| | | | 22 | ♂ | 1 | 10 | 4 | (6–8) | SGT, CA |
| | | TOTAL | 97 | | 14 | | | | |
| 2 | C3H or C3Hf | C3H | 6 | ♀ | 0 | — | 4 | (7–12) | M.T. |
| | | | 4 | ♂ | 0 | — | 0 | — | — |
| | | C3Hf | 7 | ♀ | 0 | — | 0 | — | — |
| | | | 6 | ♂ | 0 | — | 0 | — | — |
| | | TOTAL | 23 | | 0 | | | | |

* Total period of observation 16 to 20 months.
† Mice in group 1 were injected at birth with normal Ak spleen or thymus cells, thymectomized and grafted with a day-old Ak thymus. Mice in group 2 were treated as those in group 1 except that they were grafted with a day-old C3H thymus.

M.T. = mammary tumours.
SGT = salivary gland tumours }
SA = sarcoma } described in text.
CA = carcinoma }

The histological appearance of a typical lymphoid tumour arising in a thymus graft is shown in Fig 3 and 4.

None of the mice in group 2 (tolerant C3H mice bearing C3H thymus grafts) developed lymphocytic neoplasms.

The C3H females in both groups developed mammary carcinomas characteristic of the C3H strain. Eleven mice in group 1, however, unexpectedly developed a whole array of tumours which are described below. It is significant that these eleven mice were survivors from two litters injected on the same day with the same preparation of a mixture of normal Ak spleen and thymus cells, and that they were not inoculated with Passage A or other leukaemogenic filtrate.

*Immunogenetic behaviour of lymphoid tumours arising in C3H mice tolerant of Ak*

The lymphoid tumours developing in tolerant C3H mice were transplanted to both Ak mice and non-tolerant C3H adult mice (Table IV). Five were transplantable to both Ak and C3H, two grew only in Ak, and five, curiously enough, only in C3H. Four of the latter had originated when their host was 10 months old or older and one at 6 months of age, after the host had spontaneously rejected the Ak skin graft.

*Fate of lymphoid tumours in thymus homografts following adoptive immunization*

Malignancy in the subcutaneous thymus homografts became evident with the progressive enlargement of the graft. An attempt was made to abrogate tolerance in seven mice which had developed a tumour in the graft by using lymphoid cells from non-tolerant C3H mice which were previously sensitized against normal tissues (Table V). Treatment was commenced when the grafts had reached half a centimetre in the largest diameter. The tumour completely regressed in three out of the seven treated mice. This was preceded by an intense

TABLE IV.—*Transplantation of Lymphocytic Neoplasms Arising in C3H Mice Tolerant of Ak, Thymectomized and Bearing Subcutaneous Ak Thymus Grafts*

| Leukaemic donor | | | Results of transplantation by cells | | | |
|---|---|---|---|---|---|---|
| | | | In strain of origin | | In Ak strain | |
| Number | Sex | Age in months at sacrifice | Result* | Latency† (days) | Result | Latency (days) |
| 1 | ♀ | 12 | 4/6 | 21–40 | 0/6 | — |
| 2 | ♀ | 16 | 3/6 | 41–48 | 0/6 | — |
| 3 | ♂ | 5 | 0/6 | — | 6/6 | 20–30 |
| 4 | ♀ | 9 | 4/4 | 30–38 | 4/4 | 24–36 |
| 5 | ♀ | 9 | 3/3 | 34–38 | 2/6 | 36, 41 |
| 6 | ♀ | 5 | 0/4 | — | 4/4 | 18–30 |
| 7 | ♂ | 10 | 3/5 | 31–47 | 0/4 | — |
| 8 | ♂ | 8 | 3/3 | 27–31 | 3/3 | 20–25 |
| 9 | ♀ | 10 | 3/4 | 36–47 | 0/3 | — |
| 10 | ♀ | 7 | 3/5 | 20–31 | 4/4 | 22–29 |
| 14 | ♂ | 5 | 2/4 | 31, 33 | 3/4 | 26–32 |
| 15‡ | ♀ | 6 | 4/4 | 25–39 | 0/4 | — |

* Numerator = number of takes ; denominator = number of animals.

† Interval between grafting and sacrifice or death.

‡ This mouse is not included in Table III because its tumour arose after it had rejected the Ak skin graft.

The first evidence of malignancy in mice Nos. 3, 4, 5, 6, 8, 10, 14 and 15 was enlargement at the site of the thymus graft.

TABLE V.—*Effect of Immune C3H Cells on Lymphoid Tumour Growth in C3H Mice Tolerant of Ak*

| | | | Interval in days between | | Transplantation results (Table IV) | |
|---|---|---|---|---|---|---|
| Number | Sex | Age at onset of malignancy (months) | diagnosis* and 1st treatment | diagnosis and death | Takes in Ak | Takes in C3H |
| 6 | ♀ | 5 | 16 | 27 | + | − |
| 8 | ♂ | 8 | 1 | 24 | + | + |
| 10 | ♀ | 7 | 14 | 31 | + | + |
| 11 | ♂ | 5 | 18 | † | Not done | |
| 12 | ♂ | 5 | 3 | † | ,, | |
| 13 | ♀ | 6 | 1 | † | ,, | |
| 14 | ♂ | 5 | 1 | 27 | + | + |

* The day of diagnosis was arbitrarily fixed as the day when the enlargement of the graft had reached half a centimetre in its largest diameter.

† Tumour in thymus graft completely regressed from 20 to 40 days after first treatment with immune cells.

reaction in the skin graft during the second week after the first injection of immune cells. No signs of generalized leukaemia were ever present in these three mice.

The other four mice all succumbed to the disease. A feeble skin graft reaction was observed in only two and signs of dissemination of the disease became evident during the first or second week of treatment. The mice were killed when it became obvious that the treatment had failed, and the tumours were transplanted. As seen from Table IV, only one of these tumours took only in Ak. The other three were transplantable to both Ak and C3H.

*The development of parotid and other tumours in C3H mice inoculated at birth with normal Ak spleen and thymus cells*

The most unexpected result of the present experiments was the occurrence of parotid and sublingual salivary tumours, intramuscular sarcomas and other tumours in eleven C3Hf mice from two litters inoculated at birth with the same suspension of normal Ak spleen and thymus cells (Fig. 5–15). Most of the mice had salivary gland tumours on both sides (either both parotids or one parotid and one sublingual gland being involved). One mouse had three primary tumours (one parotid gland tumour, one sarcoma in the pectoral muscles and one sarcoma in the thigh muscles).

All the parotid tumours examined showed essentially the same structure. Macroscopically they were made up of discrete nodules, firm, opaque, yellowish-white and of rubbery consistency. Microscopically, a thin fibrous capsule could be seen in some places only with the growth expanding and compressing it. Outside the capsule was some lymphocytic infiltration. Two main types of structure were found in the tumour proper : duct-like structures very similar to the normal ducts of the gland (Fig. 5) and a uniform population of mesenchymal-like cells with oval nuclei and delicate cytoplasmic processes. Fig. 6 shows both these patterns while Fig. 7 shows a more solid growth in which the adenomatous pattern merges into a confluent mass of cells. Under the high power (Fig. 8) many mitoses were seen in both the duct and mesenchymal cells. There were no necrotic or degenerative changes in the tumour as a whole.

Several mice had sarcomatous tumours growing in muscle tissue. These tumours were whitish-pink solid circumscribed masses without necrosis. The histological features of two such tumours are shown in Fig. 9–11. One of the tumours was in the pectoral muscles and showed much individual cellular variation (Fig. 9 and 10). Most of the cells were large with big nuclei and a greatly increased nuclear-cytoplasmic ratio. Tumour giant cells were plentiful and mitoses frequent. Cell boundaries were hard to see in some areas and a diffuse syncytium was present. Many new blood spaces could be seen and invasion of muscle tissue was evident. No cross-striation could be found in the tumour cells when the sections were stained with phosphotungstic acid haematoxylin and the tumour was best described as un undifferentiated anaplastic sarcoma. Another sarcoma in the same mouse was growing in the muscles of the thigh. The cells were mostly fusiform (Fig. 11). The nuclei were oval and only an occasional giant cell was present. The growth could be seen invading skeletal muscle and was richly supplied with new, thin-walled, blood vessels. Fine fibrils ran between individual cells and special stains showed that the tumour cells were producing a considerable amount of collagen. The tumour was undoubtedly a fibrosarcoma.

One mouse had a bilateral parotid tumour and tumours growing in both upper eyelids (Fig. 12). These latter were excised when they reached about half a centimetre in diameter and appeared to consist mostly of fibrous tissue. Histologically, they were well-differentiated growths underlying normal epidermis. They were characterized by bundles of uniform cells running in an interlacing pattern with some palisading of their nuclei although this was not well marked. Under the high power there was more nuclear variation than would be expected for a benign neurofibroma. Also many more mitotic figures were present, suggesting the histological diagnosis of fibrosarcoma of low-grade malignancy.

One mouse had a tumour growing in the abdominal cavity. It was a spherical mass with black and red patches of necrosis and haemorrhage, attached to the upper pole of the left kidney and about the same size as the kidney itself. The left adrenal could not be found. Microscopically (Fig. 13) undifferentiated cells were seen arranged in clumps and showing numerous mitoses. Invasion of blood vessels and tumour emboli were evident in places and areas of necrosis and haemorrhage were frequent. The tumour was diagnosed as an undifferentiated anaplastic carcinoma.

No proliferative or nuclear changes were observed in the renal tubules such as has been described in mice inoculated with the polyoma virus (Stewart, Eddy and Stanton, 1959 ; Stanton *et al.*, 1959). There was an irregular lymphocytic infiltration in the cortex of one kidney, many small round cells being grouped mostly around small blood vessels. No inflammation was seen in the renal tubules nor was there any other evidence of pyelonephritis. The cells, themselves, appeared to be normal small lymphocytes (Fig. 14, 15) and no mitoses were found. There was no evidence of any leukaemic process anywhere in the animal.

Much of the above histological description was very kindly supplied by Dr. P. M. Sutton to whom I am very grateful.

### DISCUSSION

It has been observed in the experiments reported here that high-leukaemic strain Ak mice which had been made immunologically tolerant of C3H tissues and which had been thymectomized and grafted with C3H thymus tissue did not develop lymphocytic leukaemia or lymphoid tumours in the graft. These mice are said to contain a leukaemogenic agent (Gross, 1958) and some of them received, soon after birth, an injection of Passage A filtrate, which has been shown to accelerate the onset of the disease in intact Ak mice (Miller, 1960*a*). Yet, in spite of the presence in a genetically predisposed strain of both thymus tissue (genetically foreign but tolerated) and leukaemic agent, the disease failed to develop. On the other hand, low-leukaemic thymectomized C3H mice tolerant of Ak and bearing subcutaneous grafts of normal new-born Ak thymus developed lymphocytic leukaemia or malignancy in the graft as early as 5 months after birth. The mice received no injection of leukaemogenic filtrate at birth although they were injected with normal Ak cells. However, we have failed to demonstrate that such an injection of cells, *per se*, produced leukaemia in tolerant thymectomized C3H mice bearing isologous thymus grafts (Table II) or in tolerant C3H mice with intact thymuses (Miller, 1960*a*). Clearly, therefore, the genetic susceptibility to leukaemia development must depend on an intrinsic property of the thymus tissue itself. The results obtained here are in accordance with those of Law (1952, 1957) who showed that " genetically tolerant " $F_1$ hybrids from crosses between high and low leukaemic strains, bearing thymus grafts from the low leukaemic parental line, did not show neoplastic change in the grafted thymus, whereas those receiving thymus grafts from the high leukaemic parental line developed a high incidence of lymphocytic neoplasms in the graft. In similar experiments, Kaplan, Hirsch and Brown (1956) showed that C57Bl, but not C3H, thymic implants developed lymphoid tumours in irradiated thymectomized (C57Bl $\times$ C3H)$F_1$ hosts.

There are clearly two possible ways by which the presence of an Ak thymus in a tolerant C3H host could lead to the occurrence of lymphocytic neoplasms in the host. Either (1) the Ak thymus is a source of potentially malignant Ak lymphocytes which can undergo neoplastic transformation either in their own host or in a genetically foreign but tolerant host ; or (2) a non-cellular influence from the Ak thymus is responsible for the malignant change.

It would be expected on the first hypothesis that the lymphocytic neoplasms which developed in tolerant C3H mice would be transplantable to Ak mice. Two of the early neoplasms were undoubtedly of Ak origin, growing only in Ak mice, and those that regressed following injection of sensitized lymph node and spleen cells must presumably also have been of Ak origin. The behaviour of the 5 neoplasms which grew both in Ak and C3H could be explained by reference to previous work which showed that a certain percentage of Ak leukaemias would take in C3H (Furth, Boon and Kaliss, 1944) or that, by transformation or immunoselection or both, a single passage of a tumour through a tolerant foreign host allowed subsequent growth in untreated adult mice of the foreign strain (Koprowski, Gail and Love, 1956). Finally, the five neoplasms that grew only in C3H could be spontaneous C3H neoplasms that would have developed whether the Ak thymus was present or not. At least the one arising at 16 months is likely to have been a spontaneous neoplasm. The disease is, however, rare before 12 months of age and only 2 cases have been diagnosed at 12 and 14 months in 227 untreated mice observed for a period of 14 months (Miller, 1960*a*).

The second hypothesis assumes that a malignant change takes place in a population of lymphocytes, host or donor, as a result of a non-cellular influence from the thymic epithelial reticulum cells of the Ak donor. The lymphocytic population of the compatible graft (Ak) in the genetically different but tolerant host (C3H) might undergo a change, donor-type lymphocytes being gradually replaced by host lymphocytes. On the other hand, the reticular tissue of the donor thymus might survive and induce neoplastic transformation in either donor or infiltrating host lymphocytes. This may account for the fact that most of the early neoplasms were Ak in type and the later ones C3H. Again, this situation is similar to that described by Law (1952) and Law and Potter (1956). In their experiments, AKR thymus fragments increased the incidence of leukaemia in (C3Hb $\times$ AKR)$F_1$ hosts, the resulting neoplasm being transplantable only to $F_1$ hosts. In another hybrid combination, susceptible to the leukaemogenic activity of X-irradiation, malignancy developed in thymuses from unirradiated C57Bl donors grafted to irradiated (C57Bl $\times$ A)$F_1$ hosts. The tumours developing early (at about 5 months) were found to be contributed by descendants of donor C57Bl thymus tissue whereas those arising later (7–10 months) were found to have originated from $F_1$ host cells which must have populated the graft.

The transfer of immunity against homografts of skin and transplantable tumours by lymph node cells of actively immunized mice has been described by many authors (Potter, Taylor and MacDowell, 1938 ; MacDowell *et al.*, 1938 ; Brncic, Hoecker and Gasic, 1952 ; Mitchison, 1953, 1954 ; Billingham, Brent and Medawar, 1954, 1956 ; Koprowski *et al.*, 1956). By using activated lymphoid cells, Medawar and Russell (1958) showed that tolerant adrenalectomized mice making use of homografts of adrenal cortical tissue could in effect be adrenalectomized, and similar results have been obtained by Krohn (1960, personal communication) with orthotopic homografts of ovaries. In three out of seven experiments described here C3H lymphoid cells sensitized against normal Ak tissues successfully caused immunologically tolerant C3H hosts to reject normal Ak skin, and Ak thymus after neoplastic transformation. One of the tumours that failed to be rejected was transplantable only to Ak mice and it is likely that the disease was too advanced when treatment was commenced. The other three tumours that could not be made to regress presumably acquired the ability to overcome any immunity of adoptive origin in the tolerant hosts for on transplantation they grew perfectly well in both non-tolerant adult C3H hosts and in Ak mice.

The development of spontaneous leukaemia was not retarded nor was the incidence lowered in tolerant Ak mice given repeated injections of marrow or spleen cells from low-leukaemic C3H mice. A retarding effect of C3H marrow on the development of spontaneous lymphomas in (AKR $\times$ C3H)$F_1$ hybrids has been reported by Lorenz, Law and Congdon, (1954). It is possible that this effect is not demonstrable in the pure strain.

The occurrence of salivary and other tumours in tolerant C3H mice and of mammary tumours in tolerant Ak mice was unexpected. Only Ak females that had received C3H and not C3Hf cells developed breast tumours, which suggests transfer of the Bittner agent at birth via the cells. The C3Hf mice that developed salivary and other tumours all came from two litters injected on the same day with the same preparation of normal Ak cells ; all the surviving members of the two litters were affected. None received leukaemogenic filtrate. This particular distribution, the variety of the tumours, and the fact that the animals had all been injected on the first day of life with the same preparation of cells strongly suggests that the cells were obtained from Ak mice carrying the polyoma virus known to be present in various Ak stocks (Rowe *et al.*, 1959). Neither tissue culture (Stewart *et al.*, 1957) nor high speed centrifugation (Buffett *et al.*, 1958) is thus necessary to disclose the multipotentiality of this agent.

### SUMMARY

1. Acquired tolerance of C3H and Ak thymus homografts has been achieved in Ak and C3H mice, respectively, by the intravenous injection at birth of C3H and Ak thymus or spleen cells.

2. Lymphocytic neoplasms developed in Ak thymuses grafted to thymectomized tolerant C3H mice. None were, however, seen in C3H thymuses grafted to thymectomized tolerant Ak mice.

3. The lymphocytic neoplasms arising in tolerant C3H mice were in some cases transplantable only to Ak mice, in others to both Ak and non-tolerant adult C3H, and in others only to non-tolerant C3H.

4. Three out of seven lymphoid tumours developing at the site of the Ak thymus graft in tolerant C3H mice completely regressed after treatment of the host with C3H lymphoid cells from mice previously sensitized against normal Ak tissues.

5. Other tumours occurred in these experiments. Tolerant Ak female mice developed mammary carcinomas. A group of tolerant C3H mice injected at birth with the same preparation of normal Ak cells developed multiple salivary gland tumours and other tumours.

I wish to thank Professor P. B. Medawar, F.R.S. for reading the script and for his valuable advice and criticism, and Dr. L. Brent for demonstrating the techniques of intravenous injection of new-born mice and of skin grafting. Many thanks are due to Dr. N. F. C. Gowing of the Department of Morbid Anatomy, Royal Marsden Hospital, for his help in diagnosing the tumours and to Dr. P. M. Sutton of the Department of Morbid Anatomy, University College Hospital Medical School, for his description of the histopathology of these tumours. I am indebted to Professor A. Haddow, F.R.S. and to Professor P. C. Koller for their interest throughout this work and to the Gaggin Research Fellowship of the University of Queensland, Brisbane, Australia for its invaluable support. This investigation has been supported by grants to the Chester Beatty Research Institute (Institute of Cancer Research : Royal Cancer Hospital) from the Medical Research Council, the British Empire Cancer Campaign, the Jane Coffin Childs Memorial Fund for Medical Research, the Anna Fuller Fund and the National Cancer Institute of the National Institutes of Health, U.S. Public Health Service.

### REFERENCES

Billingham, R. E. and Brent, L.—(1956) *Proc. Roy. Soc.* B, **146**, 78.—(1959) *Phil. Trans.* B. **242**, 439.
*Idem*, Brent, L. and Medawar, P. B.—(1953) *Nature*, **172**, 603.—(1954) *Proc. Roy. Soc.* B, **143**, 58.—(1956) *Phil. Trans.*, B, **239**, 357.
*Idem* and Medawar, P. B.—(1951) *J. exp. Biol.*, **28**, 385.
Brent, L.—(1959) ‘ Tools of Biological Research ’, p. 57, H. J. B. Atkins, ed. Oxford (Blackwell).
Brncic, D., Hoecker, G. and Gasic, G.—(1952) *Acta Un. int. Cancr.*, **7**, 761.
Buffett, R. F., Commerford, S. L., Furth, J. and Hunter, M. J.—(1958) *Proc. Soc. exp. Biol., N.Y.*, **99**, 401.
Furth, J., Boon, M. C. and Kaliss, N.—(1944) *Cancer Res.*, **4**, 1.
Gross, L.—(1958) *Ibid.*, **18**, 371.—(1959) *Proc. Soc. exp. Biol., N.Y.*, **100**, 325.
Kaplan, H. S.—(1950) *J. nat. Cancer Inst.*, **11**, 83.
*Idem* and Brown, M. B.—(1954) *Science*, **119**, 439.
*Idem*, Hirsch, B. B. and Brown, M. B.—(1956) *Cancer Res.*, **16**, 434.
Koprowski, H., Gail, T. and Love, R.—(1956) *Proc. Roy. Soc.* B, **146**, 37.
Krohn, P. L.—(1958) *Nature*, **181**, 1671.
Law, L. W.—(1952) *J. nat. Cancer Inst.*, **12**, 789.—(1957) *Ann. N.Y. Acad. Sci.*, **68**, 616.
*Idem* and Miller, J. H.—(1950*a*) *J. nat. Cancer Inst.*, **11**, 253.—(1950*b*) *Ibid.*, **11**, 425.
*Idem* and Potter, M.—(1956) *Proc. nat. Acad. Sci., Wash.*, **42**, 160.
Levinthal, J. D., Buffett, R. F. and Furth, J.—(1959) *Proc. Soc. exp. Biol., N.Y.* **100**, 610.
Lorenz, E., Law, L. W. and Congdon, C. C.—(1954) *Ciba Symp.*, Leukaemia Research, 184, G. E. W. Wolstenholme, ed. London (Churchill).
Macdowell, E. C., Potter, J. S., Richter, M. N., Victor, J., Bovarnick, M., Taylor, M. J., Ward, E. N., Laanes, T. and Winterstein, M. P.—(1938) *Yearb. Carneg. Instn.*, **37**, 47.
McEndy, D. P., Boon, M. C. and Furth, J.—(1944) *Cancer Res.*, **4**, 377.
Martinez, C., Smith, J. M. and Good, R. A.—(1958) *Brit. J. exp. Path.*, **39**, 574.
Medawar, P. B. and Russell, P. S.—(1958) *Immunology*, **1**, 1.
Miller, J. F. A. P.—(1959*a*) *Nature*, **183**, 1069.—(1959*b*) *Ibid*, **184**, 1809.—(1960*a*) *Brit. J. Cancer*, **14**, 83 (1960*b*) *Ibid.*, **14**, 93.
Mitchison, N. A.—(1953) *Nature*, **171**, 267.—(1954) *Proc. Roy. Soc.*, B, **142**, 72.
Potter, J. S., Taylor, M. J. and Macdowell, E. C.—(1938) *Proc. Soc. exp. Biol., N.Y.*, **37**, 655.
Rowe, W. P., Hartley, J. W., Law, L. W. and Huebner, R. J.—(1959) *J. exp. Med.*, **109**, 449.
Stanton, M. F., Stewart, S. E., Eddy, B. E. and Blackwell, R. H.—(1959) *J. nat. Cancer Inst.*, **23**, 1441.
Stewart, S. E., Eddy, B. E., Gochenour, A. M., Borgese, N. G. and Grubbs, G. E.—(1957) *Virology*, **3**, 380.
*Idem*, Eddy, B. E. and Stanton, M. F.—(1959) *Acta Un. int. Cancr.*, **15**, 842.
Woodruff, M. F. A. and Boswell, T.—(1954) *Brit. J. plast. Surg.*, **7**, 211.
*Idem* and Sparrow, M.—(1958) *Quart. J. exp. Physiol.*, **43**, 91.